# Sex differences in the expression of lupus-associated miRNAs in splenocytes from lupus-prone NZB/W_F1_ mice

**DOI:** 10.1186/2042-6410-4-19

**Published:** 2013-11-01

**Authors:** Rujuan Dai, Savannah McReynolds, Tanya LeRoith, Bettina Heid, Zhihong Liang, Sattar Ansar Ahmed

**Affiliations:** 1 IDRF Building Lab 227, Department of Biomedical Sciences and Pathobiology, Virginia-Maryland Regional College of Veterinary Medicine (VMRCVM), Virginia Tech, 265 Duckpond Drive, Blacksburg, VA 24061, USA; 2 Laboratory of Food Safety and Molecular Biology, College of Food Science and Nutritional Engineering, China Agricultural University, Beijing 100083, People's Republic of China

**Keywords:** Sex differences, Lupus, microRNA, Estrogen, Splenocytes, NZB/W_F1_

## Abstract

**Background:**

A majority of autoimmune diseases, including systemic lupus erythematosus (SLE), occur predominantly in females. Recent studies have identified specific dysregulated microRNAs (miRNAs) in both human and murine lupus, implying an important contribution of these miRNAs to lupus pathogenesis. However, to date, there is no study that examined sex differences in miRNA expression in immune cells as a plausible basis for sex differences in autoimmune disease. This study addresses this aspect in NZB/W_F1_ mice, a classical murine lupus model with marked female bias, and further investigates estrogen regulation of lupus-associated miRNAs.

**Methods:**

The Taqman miRNA assay system was used to quantify the miRNA expression in splenocytes from male and female NZB/W_F1_ mice at 17–18, 23, and 30 weeks (wks) of age. To evaluate potential estrogen's effect on lupus-associated miRNAs, 6-wk-old NZB/W_F1_ male mice were orchidectomized and surgically implanted with empty (placebo) or estrogen implants for 4 and 26 wks, respectively. To assess the lupus status in the NZB/W_F1_ mice, serum anti-dsDNA autoantibody levels, proteinuria, and renal histological changes were determined.

**Results:**

The sex differences in the expression of lupus-associated miRNAs, including the miR-182-96-183 cluster, miR-155, miR-31, miR-148a, miR-127, and miR-379, were markedly evident after the onset of lupus, especially at 30 wks of age when female NZB/W_F1_ mice manifested moderate to severe lupus when compared to their male counterparts. Our limited data also suggested that estrogen treatment increased the expression of aforementioned lupus-associated miRNAs, with the exception of miR-155, in orchidectomized male NZB/W_F1_ mice to a similar level in age-matched intact female NZB/W_F1_ mice. It is noteworthy that orchiectomy, itself, did not affect the expression of lupus-associated miRNAs.

**Conclusion:**

To our knowledge, this is the first study that demonstrated sex differences in the expression of lupus-associated miRNAs in splenocytes, especially in the context of autoimmunity. The increased expression of lupus-associated miRNA in female NZB/W_F1_ mice and conceivably in estrogen-treated orchidectomized male NZB/W_F1_ mice was associated with lupus manifestation. The notable increase of lupus-associated miRNAs in diseased, female NZB/W_F1_ mice may be a result of both lupus manifestation and the female gender.

## Background

Sex differences in gene expression have physiological and pathological implications. Not only sex chromosome-linked genes but also autosomal genes are differentially expressed between males and females. It is noteworthy that sex chromosome-linked genes only count for a small number of sexually dimorphic genes; a majority of the sex differentially expressed genes are located on the autosomes since they possess about 95% of genes [1,2]. Sex differences in gene expression are regulated by a complex network comprising of sex chromosomes, sex hormones, and other sex-biased factors. Indeed, a new term, “sexome”, has been coined to encompass the sum of sex-biased effects on gene networks and cell systems [3]. Recently, much attention has been focused on the epigenetic regulation of gene expression in the context of physiological and pathological conditions. Epigenetic mechanisms such as DNA methylation, histone modification, and microRNAs (miRNAs) have been proposed to understand sex differences in gene expression and in susceptibility to a broad range of human diseases including autoimmune diseases such as lupus [1,4-7].

miRNAs are short (about 22 nucleotides long), non-coding RNAs that regulate gene expression mainly at the post-transcriptional level by binding to the 3' UTR of target genes and inducing translational inhibition or degradation of the target mRNA [8,9]. miRNAs are predicted to regulate up to 90% of human genome and thereby function as key regulators of almost all physiological systems to maintain cellular homeostasis and functions [10,11]. Thus, it is not surprising that dysregulated miRNAs have been identified and implicated in various human pathological conditions, including autoimmune-related disorders [9,12-15]. With a better understanding of the critical roles of miRNAs in disease pathogenesis, miRNAs are rapidly being considered as a new type of gene therapeutic target for treating diseases [12,16]. There are several studies that show sex differences of miRNA expression in gonadal tissues such as ovary and testis [17], and also in somatic tissues such as liver [18]. This suggests a potential contribution of miRNA regulation to the observed sexual dimorphism of gene expression and function. However, to our knowledge, thus far there is no study that has examined sex differences in miRNA expression in immune cells in the context of autoimmune disease.

It has been recognized for a long time that males and females have different immune capabilities and that they display different degrees of susceptibility to various diseases including autoimmune-related disorders [19-23]. In general, females are more susceptible to autoimmune conditions than males [19,20]. In women 65 years and younger, autoimmune diseases are among the top ten causes of all deaths and the fourth largest cause of disability [24,25]. Systemic lupus erythematosus (SLE or lupus) occurs 9–13 times more frequently in women compared to men, with over 90% of lupus patients being women. In addition to the critical contribution of genetic factors such as sex chromosome-linked genes, X chromosome monosomy, and skewed X chromosome inactivation [26,27], other factors such as hormonal and environmental factors are thought to interact with genetic factors, leading to the female prevalence of autoimmune diseases such as lupus [19,28-30]. One such mechanism by which hormonal and environmental factors regulate autoimmunity is through the regulation of miRNA expression [31].

NZB/W_F1_ is a well-characterized classical murine model for human lupus. Marked sex differences in the expression of lupus in this model are evident. Female NZB/W_F1_ mice develop moderate to severe lupus months earlier than their male counterparts. Manipulation of NZB/W_F1_ mice with sex hormones profoundly alters the disease course [32,33]. For example, administration of female hormone estrogen to male NZB/W_F1_ mice exacerbates lupus to a level that is comparable to susceptible females [33]. We recently reported that female NZB/W_F1_ mice had increased expression of lupus-associated miRNAs such as the miR-182-96-183 cluster, miR-31, miR-155, miR-127, and miR-379 only at an age when lupus is manifested [34]. miR-148a showed increased expression in female NZB/W_F1_ even before the onset of lupus [34]. The potential contribution of these individual miRNAs to autoimmune diseases has been suggested in recent studies. Among the above lupus-associated miRNAs, miR-31 and miR-148a were reported to be dysregulated in human lupus patients and contributed to human lupus pathogenesis by affecting IL-2 production and by causing CD4^+^ T cell hypomethylation and induction of autoimmune-associated genes, respectively [35,36]. miR-155 is a well-studied miRNA that plays critical roles in the regulation of both innate and adaptive immune cell development and function. miR-155 has been shown to contribute to the pathogenesis of experimental autoimmune encephalomyelitis (EAE) and rheumatoid arthritis by affecting inflammatory Th17 and Th1 cell development and function [37,38]. However, the role of miR-155 in lupus pathogenesis remains elusive [14,39]. miR-182 plays a critical role in the regulation of activated Th cell proliferation and clonal expansion, but its contribution to autoimmune disease pathogenesis is still not clear. There is also limited knowledge with regard to the immune regulatory function of miR-127 and miR-379.

In this study, we utilized both female and male NZB/W_F1_ mice to address two fundamental questions. First, given that there are marked sex differences in susceptibility to lupus, are there comparable sex differences in the expression of lupus-associated miRNAs? Second, since estrogen accelerates lupus in male NZB/W_F1_ mice, does estrogen treatment promote the expression of lupus-associated miRNAs? Our study clearly showed that female NZB/W_F1_ mice displayed increased expression levels of lupus-associated miRNAs after the onset of lupus when compared to age-matched males. Importantly, these miRNAs were disease-associated and were upregulated by estrogen treatment. Our novel findings of sexual differential expression and estrogen regulation of miRNAs in the context of lupus provide a new perspective to understand the mechanism of sex bias in autoimmune lupus.

## Methods

### Mice

Male and female NZB/W_F1_ mice (stock number 100008) were purchased from the Jackson Laboratory (Bar Harbor, ME, USA). All mice were housed in the phase IV animal facility of Virginia-Maryland Regional College of Veterinary Medicine, Virginia Tech. All procedures were approved by the Virginia Tech Institutional Animal Care and Use Committee (IACUC). The male and female NZB/W_F1_ mice were euthanized at 17/18, 23, and 30 weeks (wks) of age, respectively, and the spleen tissues were collected to isolate the splenocytes.

To determine the effect of female hormone estrogen on lupus-associated miRNA expression, per our previous published procedure [40-42], 6-wk-old male NZB/W_F1_ mice were orchidectomized and then surgically implanted with empty (placebo) or estrogen silicon implants. Considering the potential effect of orchiectomy on miRNA expression, we included a group of age-matched NZB/W_F1_ male mice that were not subjected to surgery and implant treatment (intact group). Different groups of mice were euthanized at 10 and 32 wks of age, after receiving 4 and 26 wks of implant treatment, respectively. The age-matched intact male mice were euthanized at the same time to serve as intact control.

### Splenocyte preparation

Whole splenocytes were isolated using standard lab procedures described in detail previously [34,40-43]. In brief, the spleens were dissociated by gently scraping through a steel screen, and the cell suspension was passed through a 70-μm cell strainer to remove undissociated tissue debris. The splenocytes were isolated by lysing red blood cells with ACK-Tris-NH_4_Cl buffer and then washing with complete RPMI-1640 medium (Mediatech, Inc., Manassas, VA, USA) that was supplemented with 10% charcoal-stripped fetal bovine serum (Atlanta Biologicals, Flowery Branch, GA, USA), 2 mM l-glutamine (Mediatech, Inc.), 100 IU/ml penicillin and 100 μg/ml streptomycin (Mediatech, Inc.), and 1% non-essential amino acids (Mediatech, Inc.). Freshly prepared splenocytes were pelleted and stored at -80°C for RNA isolation.

### Quantification of miRNA expression

Total RNA, containing small RNA, was isolated from the whole splenocytes using miRNeasy Mini Kit (Qiagen, Valencia, CA, USA). On-column DNase digestion with RNase-free DNase (Qiagen) was performed to remove residual amounts of DNA contamination in the isolated RNA. The RNA concentration was quantified using a NanoDrop 2000 (Thermo Fisher Scientific Inc., Wilmington, DE, USA). As we described previously [34,42], Taqman miRNA assay reagent (Applied Biosystems, Grand Island, NY, USA) was used to quantify the miRNA expression per manufacturer's instructions. The expression level of miRNA was normalized to small RNA housekeeping control snoRNA 202. The data was shown as relative expression level to an appropriate control group by using the 2^-ΔΔCt^ formula (Livak method).

### Assay of serum anti-dsDNA autoantibodies

The male and female NZB/W_F1_ mice were aged in our facility and bled retro-orbitally every 2 to 4 wks after they reached 16 or 18 wks of age. The serum anti-dsDNA antibody levels were measured by ELISA per our previous reports [34]. Briefly, the ELISA plate was coated overnight with 100 μg/ml calf thymus dsDNA (Sigma-Aldrich, St. Louis, MI, USA). After washing, the plate was blocked with PBS and 1%BSA, incubated with serum samples, followed by incubation with HRP conjugated goat-anti mouse IgG-gamma (Sigma), and lastly TMB substrate for signal development (KPL, Inc., Gaithersburg, MD, USA). The absorbance was measured by reading the plate at 380 nM with a SpectraMax M5 Microplate Reader (Molecular Devices, Sunnyvale, CA, USA).

### Measurement of proteinuria

Proteinuria was measured by dipstick analysis using Chemistrip-2GP (Roche Diagnostics Corporation, Indianapolis, IN, USA). The semi-quantitative scale was demonstrated as follows: “-”, negative or trace; “+”, 30 mg/dl; “+/-”, 30–100 mg/dl; “++”, 100 mg/dl; “++/-”, 100–500 mg/dl; and “+++”, 500 mg/dl or over.

### Renal histopathology

As previously described [34], the kidneys from the NZB/W_F1_ mice were collected and fixed in 10% buffered formalin and embedded in paraffin. Five-micron sections were stained with hematoxylin and eosin (H&E) or periodic acid-Schiff (PAS) in the histopathology lab at VMRCVM, Virginia Tech. The stained renal sections were assessed by Dr. Tanya LeRoith, a board certified pathologist, in a blinded fashion. A grade of 0 to 4 (0 = perfect, no change; 1 = minimal; 2 = moderate; 3 = marked; and 4 = severe) was given to reflect the glomerular, tubular, interstitial, and vessel inflammation and lesions, respectively. By adding the scores together, we derived an overall renal score for the microscopic changes in each sample.

### Statistical analysis

All the values in the graphs were depicted as means ± SEMs. Student *t* test and one-way ANOVA with Tukey-Kramer all pair's comparisons were performed to assess the statistical significance of miRNA expression between two groups and among the multiple groups, respectively. The JMP software (Pro10) from SAS Institute Inc. (Cary, NC, USA) was used for statistical analysis.

## Results

### Before the onset of lupus, male and female NZB/W_F1_ mice have comparable levels of lupus-associated miRNAs

In our previous study where we utilized only female NZB/W_F1_ mice, we reported that a set of miRNAs including the miR-96-182-183 cluster, miR-155, miR-31, miR-127, and miR-379 were upregulated only in the splenocytes from diseased 36–40-wk-old (8–9 months) female NZB/W_F1_ mice, but not in the splenocytes from the pre-diseased 16–18-wk-old (3–4 months) NZB/W_F1_ mice when compared to those in the control NZW mice [34]. In this study, given that there are marked sex differences in the expression and course of lupus in NZB/W_F1_ mice, we utilized both male and female NZB/W_F1_ mice to determine whether there is also sexual differential expression of aforementioned lupus-associated miRNAs.

**Figure 1:**
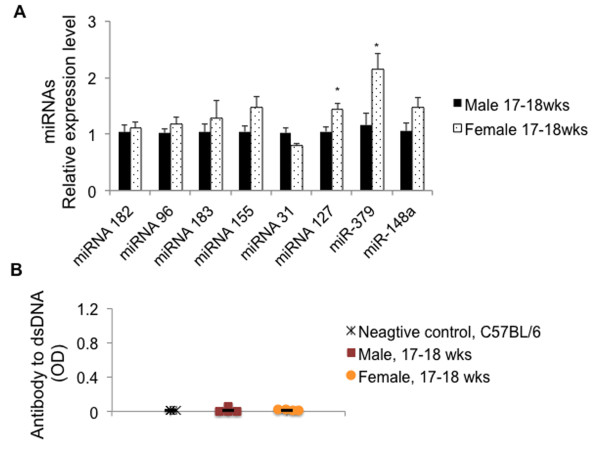
**Comparable lupus-associated miRNA levels in splenocytes from male and female NZB/W**_**F1 **_**mice before lupus onset. (A)** Real-time RT-PCR analysis of select lupus-associated miRNAs. The graph represents the means ± SEMs (*n* = 4 each). Student *t* test was performed (male vs female). *p < 0.05. **(B)** ELISA assay of serum anti-dsDNA autoantibodies. The serum samples from three non-autoimmune C57/BL6 mice were included as negative control. The mean serum anti-DNA autoantibody value in each group was indicated by *black line*.

We initially analyzed the expression of lupus-associated miRNAs including the miR-96-182-183 cluster, miR-155, miR-31, miR-127, miR-379, and miR-148a in splenocytes from male and female NZB/W_F1_ mice at 17–18 wks old, an age before the onset of disease in NZB/W_F1_ mice. As shown in Figure 1A, there was no significant difference in the expression of miR-182-96-183 cluster, miR-155, miR-31, and miR-148a between male and female mice. There was a slight, but significant increase of miR-127 and miR-379 in 17–18-wk-old female NZB/W_F1_ mice when compared to the male counterparts. As expected, there was no detection of serum anti-dsDNA antibodies in either 17–18-wk-old male or female NZB/W_F1_ mice (Figure 1B).

### After the onset of lupus, female NZB/W_F1_ mice have increased expression of lupus-associated miRNA in splenocytes compared to male counterparts

**Figure 2:**
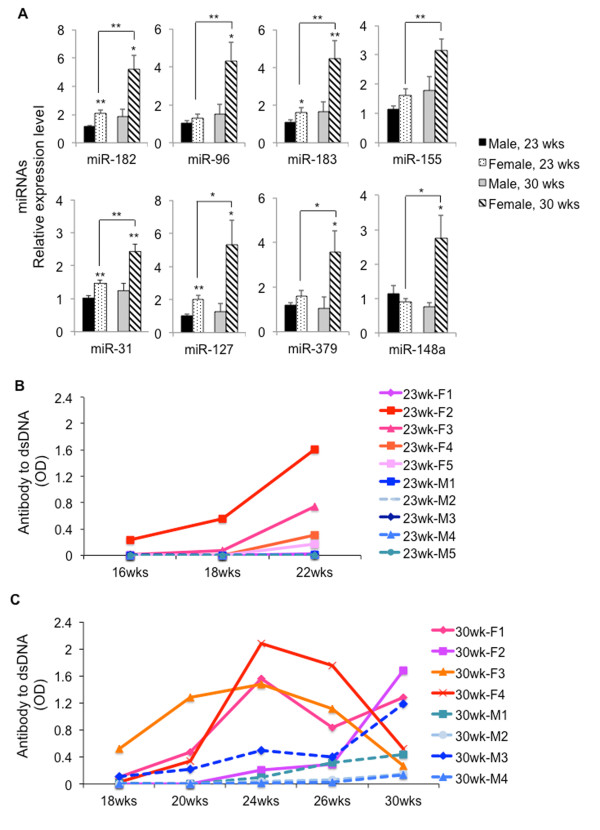
**Sex differences in the expression of lupus-associated miRNAs become evident after onset of lupus. (A)** Real-time RT-PCR analysis of lupus-associated miRNAs expression in splenocytes. The expression level of a specific miRNA in 23-wk-old female and 30-wk-old male and female NZB/W_F1_ mice was shown as the relative expression level to 23-wk-old male mice. The graphs represent the means ± SEMs (*n* = 5 each for 23-wk-old male and female mice groups, and *n* = 4 each for 30-wk-old male and female mice groups). Student *t* test was performed (age-matched male vs female, and sex-matched 23 wks vs 30 wks). *p < 0.05 and **p <0.01. **(B and C)** Anti-dsDNA autoantibody ELISA. Anti-dsDNA antibody levels were monitored sequentially every 2–4 wks in individual male (*dashed blue hue lines*) and female (*solid pink hue line*) NZB/W_F1_ mice that were terminated at **(B)** 23 wks of age and **(C)** 30 wks of age, respectively.

We next analyzed the aforementioned lupus-associated miRNAs in male and female NZB/W_F1_ mice at 23 and 30 wks of age. During this age, female, but not male NZB/W_F1_ mice, begin to develop mild to severe lupus disease. At 23 wks of age, the expression levels of miR-182, miR-183, miR-127, and miR-31 were significantly increased in female NZB/W_F1_ mice when compared to age-matched male NZB/W_F1_ mice (Figure 2A). The expression levels of these miRNAs were further increased with the exacerbation of lupus in 30-wk-old female NZB/W_F1_ mice (Figure 2A). We noticed that the expression of miR-96, miR-379, and miR-148a was significantly increased in 30-wk-old female mice, but not in 23-wk-old female mice when compared to that in age-matched male mice (Figure 2A). There was a trend of increase of miR-155 in 30-wk-old female mice when compared to age-matched male mice. It is noteworthy that we only observed a significant increase of lupus-associated miRNAs in 30-wk-old female, but not in 30-wk-old male NZB/W_F1_ mice when compared to their respective 23-wk-old, sex-matched controls. This suggests that the upregulation of these miRNAs in 30-wk-old female NZB/W_F1_ mice was not simply an aging effect, but rather was associated with lupus manifestation in female mice.

### Assessment of serum anti-dsDNA autoantibodies in male and female NZB/W_F1_ mice

To monitor lupus development and severity in NZB/W_F1_ mice, we measured the serum levels of anti-dsDNA autoantibodies (a hallmark of lupus) sequentially. Two groups of male and female NZB/W_F1_ mice were utilized in the study. The mice in the first group were sequentially bled from 16 wks of age as indicated and terminated at 23 wks of age for experimental assay (Figure 2B). The mice in the second group were sequentially bled from 18 wks of age and terminated at 30 wks of age (Figure 2C).

It is apparent that in both groups of mice, only the female NZB/W_F1_ mice demonstrated detectable levels of anti-dsDNA autoantibodies by 18 wks of age. Autoantibodies against dsDNA in the first cohort of female mice continued to increase by 23 wks of age when the mice were terminated (Figure 2B). In the second cohort of mice, the anti-dsDNA autoantibodies also continued to increase by 24 wks of age, but two females (30-wk F3 and 30-wk F4) demonstrated a sharp decline of anti-dsDNA autoantibodies in sera by 30 wks of age (Figure 2C). It is quite likely that in these two mice, the autoantibodies were directed to lupus target organs such as kidneys to induce glomerulonephritis. In support of this view, these two female mice (30-wk F3 and 30-wk F4) had a significant degree of glomerulonephritis and high level of proteinuria (over 500 mg/dl) at 30 wks of age (Table 1). In contrast to the female mice, the majority of male mice did not begin to develop anti-dsDNA autoantibodies by 30 wks of age. Overall, the female NZB/W_F1_ mice had an earlier production and higher levels of anti-dsDNA autoantibodies when compared to age-matched male counterparts.

**Table 1:** **Renal pathological changes in male and female NZB/W**_**F1 **_**mice**

	**Male 23 wks**						**Female 23 wks**				**Male 30 wks**				**Female 30 wks**			
Mouse ID	#1	#2	#3	#4	#5	#1	#2	#3	#4	#5	#1	#2	#3	#4	#1	#2	#3	#4
Glomerular score^a^	0	0	0	1	1	0	1	0	0	0	0	0	0	0	1	0	2	3
Tubular score^a^	0	0	0	0	0	0	0	1	0	0	0	0	0	0	0	0	2	3
Interstitial score^a^	0	1	0	0	0	0	0	0	1	0	0	0	0	0	0	0	2	3
Vessel score^a^	0	0	0	0	0	0	0	0	0	0	0	1	1	1	1	0	1	2
Overall renal score	0	1	0	1	1	0	1	0	1	0	0	1	1	1	2	0	7	11
Proteinuria^b^	+/-	+/-	+	-	-	+	+/-	-	-	+	+	-	-	++	++	+	+++	+++

^a^0 = perfect, no change; 1 = minimal; 2 = moderate; 3 = marked; and 4 = severe; ^b^negative or trace, -; 30, +; 30–100, +/-; 100, ++; 100–500, ++/-, 500, +++.

### Evaluation of renal pathology in male and female NZB/W_F1_ mice

**Figure 3:**
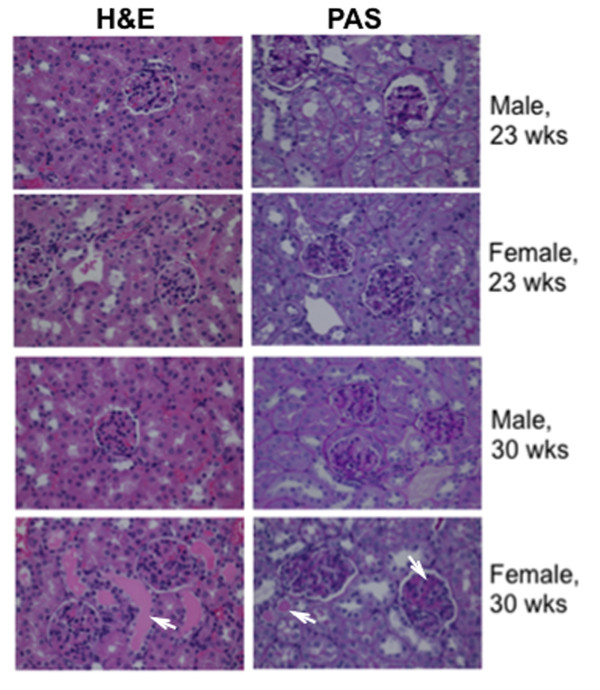
**Renal pathology of male and female NZB/W**_**F1 **_**mice.** Renal sections from 23-wk-old and 30-wk-old male and female NZB/W_F1_ mice were stained with H&E and PAS, respectively. Representative images from each age and gender group were shown. Note that only the 30-wk-old female NZB/W_F1_ mice demonstrated moderate to severe mesangioproliferative glomerulonephritis and tubular protein casts (indicated by *arrow*). The mice in the other groups showed no or minimal glomerular inflammation.

Since kidney is the major target organ for immune complex glomerulonephritis in lupus, we therefore analyzed the renal pathological changes in NZB/W_F1_ mice that were terminated at 23 and 30 wks of age. It was not surprising that there were no or only minimal histological changes in the kidneys of NZB/W_F1_ at 23 wks of age (Table 1 and Figure 3) since at this age, female mice were just beginning to develop detectable levels of anti-dsDNA autoantibodies. Conceivably, proteinuria was not detected in 23-wk-old NZB/W_F1_ mice (Table 1). By 30 wks of age, in male NZB/W_F1_ mice, there was no or minimal tissue inflammation (Table 1). In contrast to males, 30-wk-old female NZB/W_F1_ mice (30-wk F3 and F4) demonstrated moderate to marked renal inflammation and damages including membranoproliferative glomerulonephritis, protein casts, and had high levels of proteinuria (Table 1 and Figure 3). The female mouse F2, which did not develop anti-dsDNA autoantibodies until 30 wks of age, had no notable renal histological change. Taken together, our data indicated that female NZB/W_F1_ mice had earlier and more severe lupus manifestation than age-matched male counterparts.

### Estrogen regulation of lupus-associated miRNAs

**Figure 4:**
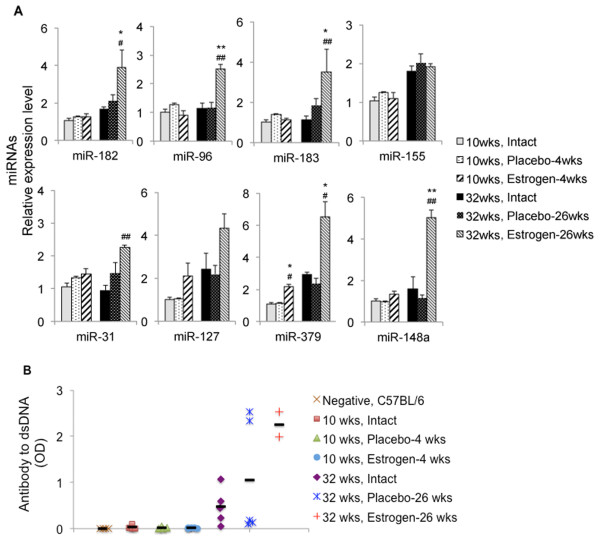
**Estrogen regulation of lupus-associated miRNAs expression in splenocytes from orchidectomized NZB/W**_**F1 **_**mice. (A)** Real-time RT-PCR analysis of miRNA expression in splenocytes from 10-wk-old and 32-wk-old intact male, placebo- and estrogen-treated orchidectomized male NZB/W_F1_ mice. The expression level of a specific miRNA in all other groups was shown as relative level to the 10-wk-old intact male mice. The graphs represent the means ± SEMs (*n* = 4 each for 10-wk-old intact and placebo-treated mice groups, *n* = 5 each for 10-wk-old estrogen-treated, 32-wk-old intact, and 32-wk-old placebo-treated mice groups, and *n* = 2 for 32-wk-old estrogen-treated mice group). One-way ANOVA with Tukey-Kramer all pair's comparison tests were performed to compare the expression among the age-matched intact, placebo-, and estrogen-treated mice. *Asterisk* indicates the statistical significance between the age-matched placebo control and estrogen-treated mice, and the *number sign* indicates the statistical significance between the age-matched intact control and estrogen-treated mice. *Asterisk* and *number sign* depict *p* < 0.05; *double asterisk* and *double number sign* depict *p* < 0.01. **(B)** Anti-dsDNA autoantibody ELISA. The figure shows the serum anti-DNA autoantibody levels in NZB/W_F1_ male mice from aforementioned six treatment groups. Four C57BL6/ mice were included as negative controls. The *bold black line* represents the mean serum autoantibody value of each group.

In our previous study, we have shown that estrogen regulates inflammatory responses by modulating the expression of selected miRNAs in splenocytes of orchidectomized wild-type C57BL/6 (B6) male mice [42]. Since estrogen has been well documented to exacerbate lupus, especially in NZB/W_F1_ murine model [32,33], we investigated whether estrogen regulates lupus-associated miRNAs expression in NZB/W_F1_ mice. We examined estrogen's effect on miRNA expression after 4 and 26 wks of estrogen treatment (the mice were 10-wk-old and 32-wk-old, respectively, at the time when they were terminated). As shown in Figure 4A, there was no significant increase of most lupus-associated miRNAs in 10-wk-old estrogen-treated orchidectomized mice when compared to age-matched intact or placebo-treated orchidectomized mice. The only exception is miR-379, which was increased in the splenocytes from 10-wk-old estrogen-treated mice. miR-182-96-183, miR-379, and miR-148a were significantly increased in 32-wk-old estrogen-treated mice when compared to either age-matched intact or placebo-treated mice. There was a trend (albeit not significant) of increase of miR-31 and miR-127 expressions in 32-wk-old estrogen-treated mice when compared to 32-wk-old placebo-treated control mice. Interestingly, estrogen treatment did not affect the expression of miR-155. Together, our data indicate that estrogen treatment promoted the expression of lupus-associated miRNAs with the exception of miR-155 in splenocytes from NZB/W_F1_ mice. It is noteworthy to point out that orchiectomy itself did not affect the expression of lupus-associated miRNAs since there was no difference in the expression levels of these miRNAs between placebo-treated orchidectomized and intact control mice.

### Assessment of autoimmune parameters in placebo- and estrogen-treated orchidectomized male NZB/W_F1_ mice

We measured the serum anti-dsDNA antibody levels in all the orchidectomized mice that were subjected to placebo or estrogen treatment for 4 or 26wks. The age-matched intact control males were assessed at the same time. As expected, there was no detection of anti-dsDNA autoantibodies in all 10-wk-old NZB/W_F1_ mice with or without implant treatment (Figure 4B). In 32-wk-old intact male NZB/W_F1_ mice, we detected moderate levels of anti-dsDNA antibodies, which were similar to what we have observed in 30-wk-old NZB/W_F1_ male mice. In contrast, 32-wk-old estrogen-treated mice had noticeable higher levels of anti-dsDNA autoantibodies (Figure 4B). For placebo-treated NZB/W_F1_ mice, three mice had basal levels of anti-dsDNA autoantibodies, and the other two mice had high levels of anti-dsDNA antibodies (Figure 4B). We noticed that individual mice from 32-wk-old intact, placebo-, and estrogen-treated groups had minimal to moderate renal inflammation and glomerulonephritis (Table 2). Even though two placebo-treated mice had high levels of anti-dsDNA autoantibodies that were comparable to estrogen-treated mice, there was no marked increase of lupus-associated miRNA expression in these two placebo-treated mice. We only observed a significant increase of lupus-associated miRNAs in estrogen-treated orchidectomized NZB/W_F1_ mice. This suggests that enhanced anti-dsDNA autoantibody levels do not closely reflect the increase of lupus-associated miRNAs. The increase of lupus-associated miRNAs in estrogen-treated mice might be due to a combination of disease manifestation and estrogen effect.

**Table 2:** **Renal evaluation of 32-wk-old intact, placebo-, and estrogen-treated orchidectomized NZB/W**_**F1 **_**mice**

	**Intact 32 wks**					**Placebo 32 wks**					**Estrogen 32 wks**	
Mouse ID	#1	#2	#3	#4	#5	#1	#2	#3	#4	#5	#1	#2
Glomerular score^a^	1	2	0	0	0	1	1	0	0	2	0	2
Tubular score^a^	1	1	1	1	2	1	1	1	1	0	1	1
Interstitial score^a^	0	0	0	0	0	2	0	0	0	0	0	1
Vessel score^a^	1	0	0	0	0	1	1	0	0	2	0	1
Overall renal score	3	3	1	1	2	5	3	1	1	4	1	5
Proteinuria^b^	+/-	+/-	-	+	+/-	+/-	++	+/-	+/-	+	-	++/-

^a^0 = perfect, no change; 1 = minimal; 2 = moderate; 3 = marked; and 4 = severe; ^b^negative or trace, -; 30, +; 30–100, +/-; 100, ++; 100–500, ++/-, 500, +++.

### Time effect of estrogen treatment on miRNA expression

**Figure 5:**
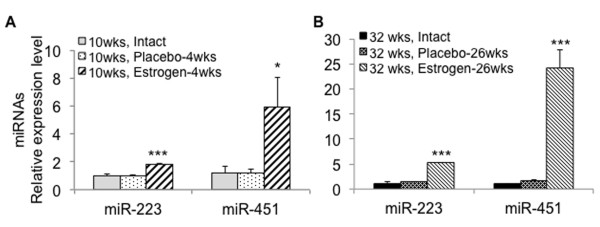
**Estrogen increased the expression of miR-223 and miR-451 expression in splenocytes from NZB/W**_**F1 **_**mice.** The graphs show increased miR-223 and miR-451 expression in splenocytes from 10-wk-old **(A)** and 32-wk-old **(B)** estrogen-treated orchidectomized NZB/W_F1_ mice when compared to either age-matched placebo control or intact control mice. The graph shows the means ± SEMS (*n* = 2 for 32-wk-old, estrogen-treated mice group, and *n* = 4 each for the other groups). Student *t* test was performed (estrogen vs placebo or intact). *p < 0.05 and *** *p* <0.001.

In this study, we observed that short-term estrogen treatment (4 wks) did not increase miR-148a (Figure 4A), which has been shown to be upregulated by estrogen in orchidectomized wild-type B6 mice [42]. One possible explanation is that 4 wks of estrogen treatment was not sufficient to alter the expression of the miRNAs that were analyzed in this study. To confirm the time dependence of estrogen effect on miRNA expression in NZB/W_F1_ mice, we analyzed the expression of miR-223 and miR-451, which were markedly upregulated by estrogen in wild-type B6 mice [42]. As expected, the expression levels of miR-223 and miR-451 were significantly upregulated in both 10-wk-old (Figure 5A) and 32-wk-old (Figure 5B) estrogen-treated mice when compared to age-matched intact or placebo-treated mice. Unlike lupus-associated miRNAs such as the miR-182 cluster that was highly increased in diseased, 36–40-wk-old NZB/W_F1_ mice [34], miR-223 and miR-451 were not significantly increased in 36–40-wk-old female NZB/W_F1_ mice when compared to pre-diseased NZB/W_F1_ or NZW control (Additional file 1: Figure S1). This suggests that the remarkable increase of miR-223 and miR-451 in 32-wk-old estrogen-treated mice was attributable to estrogen effect, but not to lupus manifestation. It is noteworthy that the magnitude of miR-223 (about 2-fold) and miR-451 (about 6-fold) increase in 10-wk-old estrogen-treated NZB/W_F1_ mice was further increased in 32-wk-old estrogen-treated NZB/W_F1_ (over 5-fold for miR-223 and over 20-fold for miR-451) when compared to those in intact controls (Figure 5). This data further suggested a potential time effect of estrogen treatment on the expression of miRNAs.

**Figure 6:**
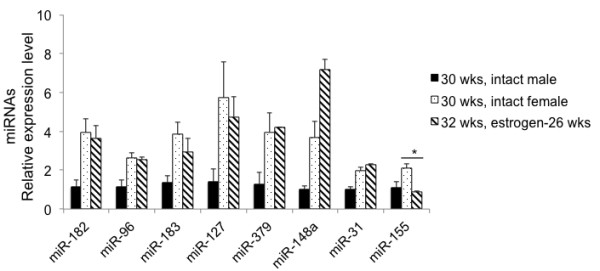
**Comparable lupus-associated miRNAs levels in splenocytes from estrogen-treated male and age-matched intact female NZB/W**_**F1 **_**mice.** The expression levels of miRNA in 30-wk-old intact female and 32-wk-old estrogen-treated orchidectomized NZB/W_F1_ mice were shown as the relative expression levels to 30-wk-old intact male mice. The graph shows the means ± SEMS (*n* = 2 for 32-wk-old estrogen-treated mice group, and *n* = 4 each for 30-wk-old intact male and female mice groups). Student *t* test was performed (30-wk-old female NZB/W_F1_ vs 32-wk-old estrogen-treated orchidectomized NZB/W_F1_). *p < 0.05.

We then compared the expression level of lupus-associated miRNAs in 32-wk-old, estrogen-treated orchidectomized male NZB/W_F1_ mice with that in intact female NZB/W_F1_ mice at a similar age (30 wks old, Figure 6). Impressively, the expression levels of lupus-associated miRNAs in estrogen-treated orchidectomized male NZB/W_F1_ mice were comparable to those in 30-wk-old female NZB/W_F1_ mice. The only exception was miR-155, which was not regulated by estrogen treatment (Figure 4). Overall, our data indicated that estrogen treatment promoted the expression of lupus-associated miRNAs (except miR-155) in NZB/W_F1_ male mice to a similar level as that in age-matched female mice. This suggests that estrogen-induced acceleration of lupus in male NZB/W_F1_ mice might be associated with enhanced expression of select lupus-associated miRNAs.

## Discussion

The sexual dimorphisms of genome structure and gene regulation imply the sex difference in susceptibility to human disease such as autoimmune disease, which disproportionally affect women [7]. There is now a flurry of recent reports documenting the critical role of non-coding miRNAs in the control of autoimmunity [9,14,15,44,45]. However, thus far, there is no study that addresses whether there are sex differences in miRNA expression as a plausible explanation for the sex bias of autoimmune diseases. In this study, we examined whether previously identified lupus-associated miRNAs are differentially expressed in gonadal intact male and female NZB/W_F1_ mice. Considering that estrogen plays an important role in the female prevalence and severity of murine lupus, we further investigated whether estrogen augments the expression of lupus-associated miRNAs in NZB/W_F1_ mice.

Impressively, we found that after the onset of lupus, the expressions of lupus-associated miRNAs (miR-182-96-183, miR-31, miR-127, miR-379, and miR-148a, miR-155) were significantly increased in female NZB/W_F1_ mice when compared to those in age-matched male mice. The sex differences in the expression of the above miRNAs became more prominent at 30 wks of age when moderate and marked lupus was evident in the female NZB/W_F1_ mice (Figure 2A). The autoimmune statuses were confirmed in 30-wk-old female NZB/W_F1_ mice as indicated by the increased anti-dsDNA autoantibodies (Figure 2C), proteinuria, and marked renal pathological changes when compared to those in male counterparts (Figure 3 and Table 1). It is noteworthy that the expression levels of most lupus-associated miRNAs in pre-diseased (17–18 wk old) female NZB/W_F1_ mice were comparable to those in male counterparts (Figure 1). Further, our findings tend to support the proof of principle that estrogen can augment lupus-associated miRNAs (with the exception of miR-155) in relatively resistant males (Figure 4).

Our finding of increased miR-182 cluster, miR-155, miR-31, and miR-148a expression in female NZB/W_F1_ mice at an age after the onset of lupus validates our previous report of the association of these miRNAs with lupus manifestation in this model. Thus far, the potential contribution of the miR-182-96-183 cluster to lupus remains elusive. miR-182 is substantially increased in activated T cells, which is critical for the proliferation and expansion of activated T helper (Th) cells [46]. The direct link between miR-182 and autoimmune inflammation is not definitive thus far. However, its role in autoimmunity is suggested by the finding that the inhibition of miR-182 in T cells reduced ovalbumin (OVA)-induced arthritis in mice [46]. Moreover, our unpublished data revealed that the inhibition of this miRNA cluster in murine lupus splenocytes significantly depressed the production of inflammatory cytokines that are implicated in lupus pathogenesis. miR-148a was the only miRNA that displayed the increased expression in pre-diseased female NZB/W_F1_ mice when compared to those in NZW controls [34]. Its expression was further increased in 36–40-wk-old female NZB/W_F1_ mice accompanying lupus progression (unpublished data). The finding of increased miR-148a in female mice at 30 wks of age is significant since miR-148a was reported to be highly upregulated in both human and murine lupus T cells, which contributed to DNA hypomethylation and induction of autoimmune-associated genes by targeting DNA methyltransferase 1 (DNMT1) [36]. We observed an increase of miR-31 in splenocytes from murine lupus, which may contribute to autoimmunity by suppressing regulatory T cell (Treg) development or function as it was reported to target Foxp3, a lineage specific transcription factor for Tregs [47]. Unlike our finding in murine lupus models, human lupus peripheral blood T cells had decreased miR-31 expression, which correlated with reduced IL2 production in human lupus T cells [35].

Of the eight lupus-associated miRNAs analyzed in this study, only miR-127 and miR-379 displayed sexual differential expression before the onset of lupus in NZB/W_F1_ (Figure 1). Interestingly, we found that only miR-379 was increased in splenocytes from 4 wks of estrogen-treated orchidectomized NZB/W_F1_ mice when compared to placebo-controls (Figure 4A). miR-127 and miR-379 are encoded by a large miRNA cluster embedded in the mouse maternal imprinted region Dlk1-Gtl2 and human homologues DLK1-DIO3. Impressively, our previous microarray data indicated that in addition to miR-127 and miR-379, several other miRNAs from the Dlk1-Gtl2 region, including miR-433, miR-300, and miR-382, were also increased in MRL-lpr and B6-lpr mice [34]. So far, there is limited knowledge about Dlk1-Gtl2 imprinted miRNA clusters such as miR-127 and miR-379 with regard to their function, especially their immune regulatory function. Further investigation is warranted to understand the contribution of Dlk1-Gtl2 (or human DLK1-DIO3) genomic imprinted miRNAs to autoimmune disorders.

Even though the effect of estrogen on human lupus is still debatable, estrogen undoubtedly induces earlier onset of lupus and exacerbates lupus severity in murine lupus models, especially in NZB/W_F1_[32,33]. We have reported that estrogen-modulated miRNA expression in orchidectomized wild-type B6 mice [42]. Importantly, estrogen-downregulated miRNAs such as miR-146a and miR-125 were also shown to be depressed in PBMC or CD4^+^ T cells from human lupus patients and inversely correlated with lupus activity [44,48]. The estrogen-upregulated miRNA miR-148a was increased in CD4 T cells from human lupus patients and positively correlated with lupus activity [49]. This suggested a potential effect of estrogen on lupus by regulating the expression of lupus-associated miRNAs.

Previous reports have documented that pre-pubertal castration and estrogen treatment of male NZB/W_F1_ mice greatly enhanced mortality (60% by age of 28 wks old) [33]. In our study, we lost about 55% of estrogen-treated mice (five out of nine mice) by the age of 32 wks. At this age, all mice belonging to the intact control and placebo-treated groups survived. Among the four estrogen-treated mice that survived to 32 wks of age, two mice developed severe lymphoma in addition to lupus (referred to as estrogen-lymphoma). The other two estrogen-treated mice without lymphoma development were referred to as estrogen-“normal” to distinguish them. The two surviving estrogen-lymphoma mice displayed distinct and different pathological changes that were not usually observed after estrogen treatment. One had markedly enlarged spleen, thymus, and liver, and the other had a hardened thymus and stomach knot. Since these pathological changes and lymphoma development were not expected in our vast experience with estrogen-treated mouse model, we excluded these two estrogen-lymphoma mice in the main body of study. Nonetheless, the data from the estrogen-lymphoma mice was analyzed separately (please see Additional file 1: Figure S2 for details). Despite the lymphoma development and other pathological changes, these two estrogen-lymphoma mice displayed similar increases for some lupus-associated miRNAs such as miR-182-96-183 as estrogen-normal mice. However, these two estrogen-lymphoma mice displayed large variations in expression of other miRNAs such as miR-127, miR-327, and miR-31 (Additional file 1: Figure S2). We recognized that the limited sample size of estrogen-treated mice does not allow us to draw firm conclusions. However, our data tends to support that estrogen treatment promoted the expression of selected lupus-associated miRNAs such as the miR-182 cluster, miR-379, and miR-148a in orchidectomized male NZB/W_F1_ mice.

Previous reports have investigated the sexual dimorphism of protein-coding gene expression and the effect of sex hormones in the regulation of protein-coding genes with focus on immune response genes in both human and mouse immune cells [50-52]. In this study, we are the first to show sex differences in the expression of small, non-protein-coding RNAs (miRNAs) in lupus-prone NZB/W_F1_ mice. Further integration of sexually dimorphic miRNA and messenger RNA (mRNA) gene expression data together should provide us a comprehensive understanding of the molecular mechanism of sex differences in immune function and autoimmune disease susceptibility. In addition, further in-depth investigation of the causative relationship between dysregulated lupus-associated miRNA expression and lupus manifestation is warranted to understand the pathogenic contribution of miRNAs to autoimmune lupus, which is still in its infancy stage.

## Conclusions

The core purpose of this study was to address whether or not there are sex differences in the expression of lupus-associated miRNAs. To this end, our study clearly demonstrated the existence of sexual dimorphism of lupus-associated miRNA expression in NZB/W_F1_ mice, which was more evident after the onset of lupus. To our knowledge, this is the first report of sexually dimorphic expression of lupus-associated miRNAs in the context of autoimmune lupus. Further, our initial study also demonstrated that estrogen exposure accelerated the expression of select lupus-associated miRNAs in castrated male NZB/W_F1_ mice, thereby conferring male NZB/W_F1_ mice a female expression pattern of these miRNAs. These novel observations provide us new insight into the role of sexually dimorphic miRNA expression in the sex bias of autoimmune diseases.

## Competing interests

The authors declare that they have no competing interests.

## Authors’ contributions

SAA and RD are the PIs of this study, conceived and executed the study, interpreted the data, and drafted the manuscript. SM, BH, and ZL assisted in sample collection, performed the experiments, and assisted in data analysis. SM helped in editing the manuscript. TL performed the evaluation of renal histopathology changes and assisted in data interpretation. All authors read and approved the final manuscript.

## Authors’ information

SAA is a Professor of Immunology and the Head of the Department of Biomedical Sciences and Pathology (DBSP), Virginia-Maryland Regional College of Veterinary Medicine (VMRCVM), Virginia Tech. RD is a Research Assistant Professor of Immunology at DBSP, VMRCVM, Virginia Tech. SM is a DVM student at VMRCVM, Virginia Tech. TL is a Clinical Associate Professor of Anatomic Pathology and a board-certified Veterinary Pathologist at DBSP, VMRCVM, Virginia Tech. BH is a Research Laboratory Specialist in the laboratory of DBSP, Virginia Tech. ZL is an Associated Professor at China Agricultural University (CAU) and was a visiting research scholar at DBSP, Virginia Tech.

## Supplementary Material

Additional file 1 **Supplemental data.** The expression of miR-223 and miR-451 in splenocytes was not increased with lupus manifestation in NZB/W_F1_ mice **(Figure S1)** and the diversity in the expression level of select lupus-associated miRNAs in estrogen-treated mice with lymphoma development **(Figure S2)**. Click here for file
