# Supplementary material for: Sex differences in the expression of lupus-associated miRNAs in splenocytes from lupus-prone NZB/WF1 mice
Source: Biol Sex Differ. 2013 Nov 1;4:19. doi: 10.1186/2042-6410-4-19 (PMC3843556; doi:10.1186/2042-6410-4-19)
Supplement: Additional file 1 — Supplemental data. The expression of miR-223 and miR-451 in splenocytes was not increased with lupus manifestation in NZB/WF1 mice (Figure S1) and the diversity in the expression level of select lupus-associated miRNAs in estrogen-treated mice with lymphoma development (Figure S2). [file 2042-6410-4-19-S1.docx]

**Supplemental Data**

**
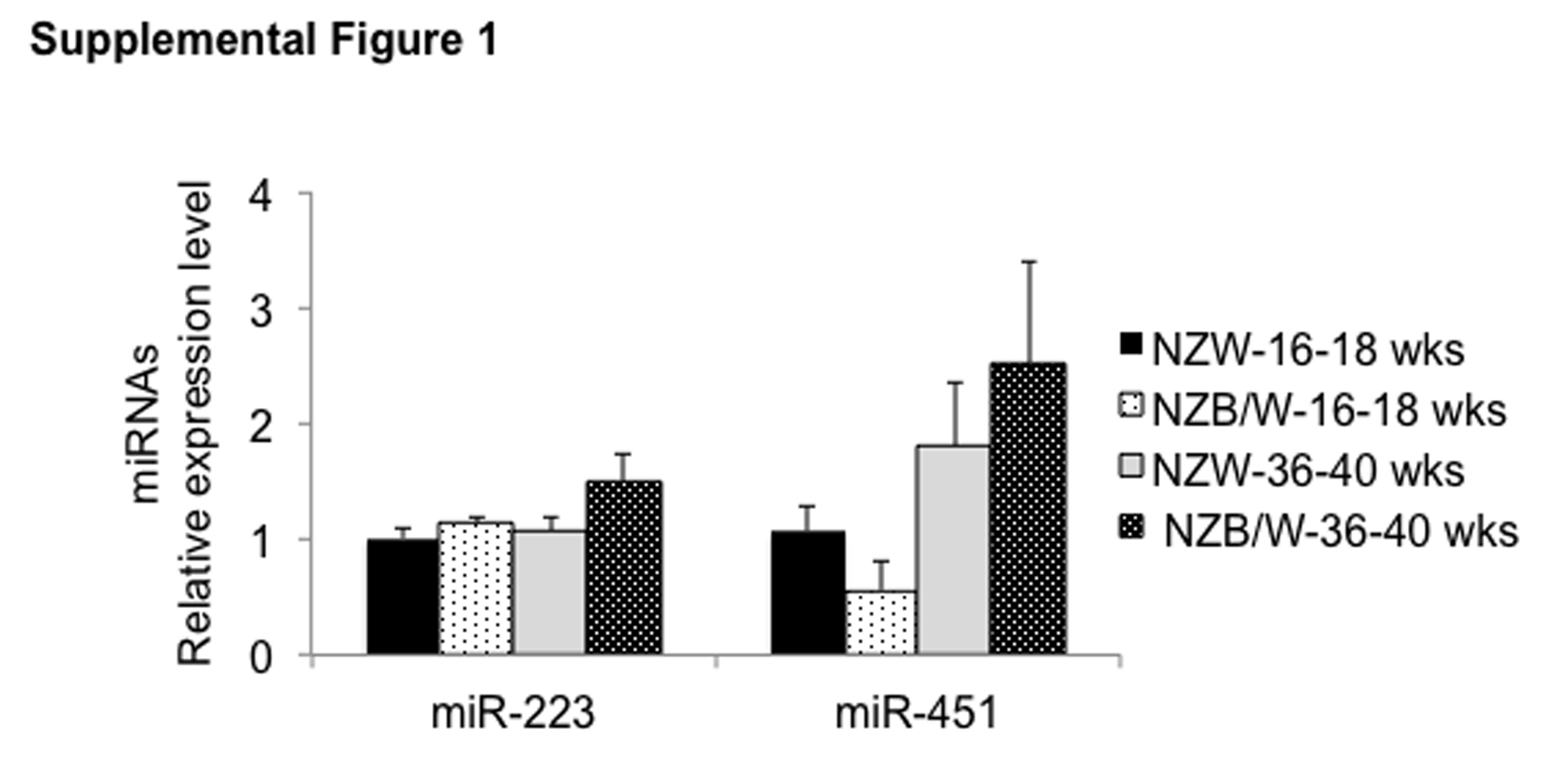
**

**Supplemental Figure 1: The expression of miR-223 and miR-451 in splenocytes was not significantly increased in diseased, 36-40 wk old female NZB/W_F1_ mice when compared to pre-diseased, 16-18wk old NZB/W_F1_ mice or NZW control mice.** The graph shows means ± SEM (n=4 each).


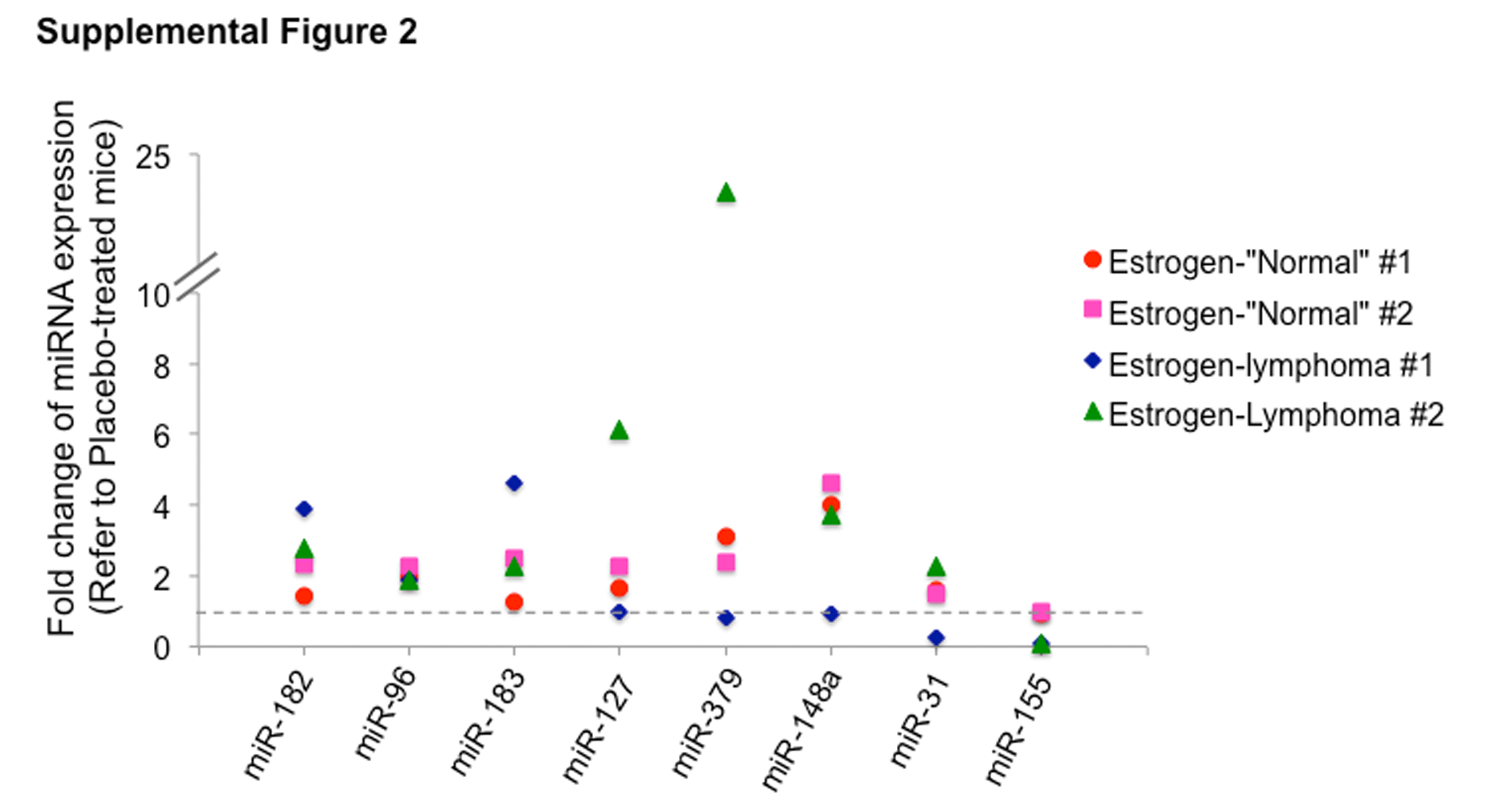


**Supplemental Figure 2: Diversity in the expression level of select lupus-associated miRNAs in estrogen-treated mice with lymphoma development.** The graph shows the expression levels of lupus-associated miRNAs in individual 32 wk old, estrogen-treated mice as the fold change to age-matched placebo control mice. The reference value was marked by dashed line. Estrogen-treated mice that developed lymphoma were referred to as Estrogen-lymphoma. The other two mice that did not have lymphoma were referred to as estrogen-“normal”. In all estrogen-treated mice, regardless of lymphoma development or not, there was a comparable increase of the miR-182-96-183 cluster. Although miR-127, miR-379, miR-148a and miR-31 were increased in estrogen-lymphoma #2, they were not increased and even reduced (for miR-31) in estrogen-lymphoma#1 when compared to placebo controls. While miR-155 was not changed in estrogen-“normal” mice, it was dramatically reduced in both estrogen-lymphoma mice when compared to placebo-treated control mice.
